# On Syphilis, Constitutional and Hereditary, and on Syphilitic Eruptions

**Published:** 1852-11

**Authors:** 


### BIBLIOGRAPHICAL NOTICES.

*On Syphilis, Constitutional and Hereditary; and on Syphilitic Eruptions.* By ERASMUS WILSON, F. R. S., Author of "A Treatise on Diseases of the Skin," etc. *With Four Colored Plates.* Philadelphia: Blanchard and Lea, 1852. 8vo. pp. 284.

This book has considerable pretensions on the score of originality, the author having, he states, "confined himself strictly to his own experience, and limited his remarks to observations made with his own eye." His work appears "as an arranged series of cases, representing the subjects of his inquiry, and the sources of his conclusions." He particulararly claims to have placed the subject of syphilitic eruptions "upon a more correct basis than that on which they had previously stood," and attempts to establish that there exists in fact "but one *eruption*, and that the apparent differences in the character of the cutaneous affection [of syphilis] are the simple consequence of modification of development of that eruption, a modification depending for the most part, on time, treatment, and the temperament of the patient."

Dr. Wilson's views on the general subject of syphilis appear to us in the main sound and judicious, and we commend the book as an excellent monograph on the subject. But, while we are prepared to go very far with him, in estimating the consequences of the syphilitic contamination upon the human system, we cannot but consider that he has much overcharged the picture of its effects, and has been led into great exaggerations. It is certainly going far to assert, "that the tenacity of the syphilitic poison to the human organization cannot but lead to the conclusion that, once admitted into the blood and tissues of the body, it remains there for life. It may not manifest its presence by any outward sign, but this cannot be received as an argument against its existence; . . . . . . . . and should the individual escape, his children may suffer sooner or later; and I am firmly of opinion, that the powers of the poison may be manifested after the lapse of several generations." And, again, "the poison of syphilis is of a most enduring kind; once received into the blood, it remains there for years, and possibly—indeed certainly—for the rest of existence." "A person possessed of this poison is capable of conveying it to another; and if that other be his wife, he may, through her means, convey it also to his child." "The infected wife must remain infected as long as she continues to live; and, by a parallel reasoning, the infected child must remain infected until death!" There is something really appalling in these doctrines, involving, as they almost do, the final *syphilisation* of the whole human race. And, indeed, they are apparently contradicted by the author himself, who, in his chapter on treatment, certainly implies his frequent successful elimination of the syphilitic virus from the human system.

He traces, also, to "that widely spread, that almost universal, animal poison, syphilis," various disorders not usually connected with it. "Lupus, Kelis, and Lepra and Psoriasis are forms of cutaneous disease, all having their original source in syphilis, all maintaining a relationship in different degrees of remoteness with that disease, and all, therefore, falling into the category of hereditary syphilis." And scrofula, too, is assumed to owe its orign to the syphilitic poison." "Is NOT LUPUS, SYPHILIS?" Dr. Wilson asks. "Lupus is scrofula; and what, we might ask, is scrofula? Is SCROFULA, SYPHILIS?" And, in another place: "A considerable portion of those diseases, which pass under the name of Scrofula, are the produce of the syphilitic poison—are, in fact, not scrofulous, but syphilitic." In short, we rise from the book, almost with a doubt if every thing morbid is not syphilis, and all mankind doomed to the inevitable contamination.

Notwithstanding, however, this disposition to overstrain his subject, Dr. Wilson has presented us a very faithful and lucid description of syphilis, and has cleared up many obscure points in connection with its transmissibility, pathology, and sequelæ. His facts and inferences will, we are satisfied, be received as decisive in regard to many *questiones vexatæ.* They appear to us entitled to notice at some length.

First, with respect to the *transmission* of the syphilitic poison. Dr. Wilson thinks that it may be communicated by the natural secretions of a person who has been contaminated, though, "to all appearance cured of the consequent diseases,"—"without the presence of a primary disease in the infector, or the intervention of a primary disease in the infectee." Numerous cases —many very strong ones—are detailed in apparent confirmation of this view. As far as regards the transmissibility of actually existing constitutional syphilis, our opinions are in accordance with the author's. And we believe him also to be right in the subjoined speculation:—

"The recognition of the contagion of constitutional syphilis, will go far to explain a circumstance which must have fallen under the observation of every unprejudiced investigator of the syphilitic poison and its manifestations, namely, the occurrence of syphilitic eruptions and other symptoms of constitutional syphilis after gonorrhœa. When we see a man perfectly free from any primary symptoms of disease, communicating syphilis to his newly-married wife, by his secretions alone, can we doubt the possibility of a similar result accruing from a syphilitic secretion poured out by the mucous membrane, as happens in gonorrhœa? I do not say that every gonorrhœa is syphilitic; on the contrary, I know that few are so, but those few have as much the power of transmitting syphilis as an undoubted chancre. I am led to make this observation, because, in numerous instances of syphilitic eruptions which have come under my notice, I have been told that the patient has never suffered from any other form of venereal disease than gonorrhœa, and this has been used as an argument against the possibility of a resulting constitutional syphilis. It is an argument which should be received with distrust."

In connection with primary syphilis, we may note the author's opinion, that sloughing and phagedænic chancres are rarely followed by constitutional symptoms, "the excessive local action which occurs in them seeming to destroy the virus-forming process which would have otherwise have been set up. The calm and natural action present in the simple chancre, being favorable to development of the virus, renders it more dangerous as a source of constitutional infection." The indurated chancre "is invariably followed by constitutional syphilis; indeed is itself a manifestation of constitutional action; in other words, the induration is a constitutional affection superadded to the primary disease."

The syphilitic poison, if introduced into the blood, excites in that fluid an action "which results in the ejection or elimination of the poison."

"The symptoms which may be selected as pathognomonic of syphilitic fever are: mental and nervous depression and prostration; congested fauces, with sore-throat; congested and muddy conjunctiva; congested and discolored skin, the congestion being partial or general, and assuming the form of an eruption; and, added to these, neuralgic pains."

In this combination of symptoms the author sees a resemblance to the fevers usually termed exanthematous, . . . . the syphilitic fever being considered a *chronic* exanthema, while the others are *acute.*

In the effort on the part nature to eject the noxious poison from the blood, the superficial membranes chiefly suffer.

"The syphilodermata, or syphilitic eruptions of the skin, present two principal forms, the one being simply congestive and unattended with elevation of the skin; the other, presenting the obvious feature of elevation. To the *non-elevated* group belong roseola, syphilitic maculæ, and erythema; to the *elevated* group, the small pimples of lichen and the larger pimples or tubercles of tubercular syphilis. These differences are, however, more apparent than real, and may be regarded as stages of development of the same disease. Roseola, by an easy gradation, is converted into lichen, or tubercular syphilis, and these latter, by simple subsidence, become syphilitic maculæ.

The period, at which the roseolous rash makes its appearance, is between six and nine days after the development of the primary disease. The lichenous eruption occurs with more irregularity as to the period of outbreak,—usually in delicate subjects or under circumstances of exposure. "The tubercular eruption differs from lichen only in the size of the little elevations which give it its specific characters."

As regards the "copper color" of syphilitic eruptions, according to Dr. Wilson,

"The copper color represents in fact, a declining stage of the eruption, when the congestion is subsiding, and the yellow stain of the altered fluids of the skin shines through the purple of the blood. The 'copper color,' therefore, may have a greater or less amount of red or yellow in its composition, and be either a reddish copper color or a yellowish copper color.

As the copper color represents only a stage of the eruption, that eruption having probably passed through the tints of dull-red, and dull purplish-red, before it reaches the reddish-yellow brown of copper color, it is clear that the term is objectionable when taken as a pathognomonic sign of a syphilitic eruption. For, if we see the eruption at any other period than that of its decline, the characteristic tint is absent."

Numerous varieties are described of tuberculous syphilis.

As regards vesicular syphilis, the author has "never seen a case of constitutional syphilis deserving of a place under this head." Indeed, he can hardly conceive the eruption of syphilis to assume the character of eczema or herpes.

"The only eruption coming strictly under the denomination of *pustular* syphilis, is rupia. . . . Other forms of pustule must be considered as instances of suppurating papules and tubercles."

Rupia forms "a natural link of transition from the superficial ulcerations of syphilitic tubercles, to those deep, unhealthy, and often sloughing ulcers, which may be distinguished, *par excellence*, as syphilitic ulcers."

Syphilitic tumors, (*tubercula gummata*,) Dr. Wilson considers to result from the modification of syphilis by time.

"They seem to deserve a place by themselves under the title of 'chronic syphilis;' or, if it be preferred, tertiary syphilis. In their hardness they remind us of cancer, and are very likely to be mistaken for that disease. When they ulcerate, ulceration takes place very slowly, and generally on one side, while by the other they continue to grow; hence the ulcer has more or less of a horse-shoe form, and the tissues over which it has passed heal, but leave an indelible cicatrix. The ulcer is slowly destructive, and exhibits no tendency to granulate; sometimes it dissects out certain tissues with great neatness."

Besides these general effects of the syphilitic poison on the skin, the author describes many others, which are local or partial; "such actions being either present as symptoms among the general phenomena, or having an independent existence." They are alopecia and other affections of the hair, affections of the nails, affections of the hands and feet, (termed by him erythema palmare et plantare, though "generally going by a name to which they are by no means entitled, psoriasis,") condyloma, affections of the mucous membranes, affections of the tongue, affections of the nervous system, and, lastly, of the periosteum, ("much more rare at the present day than in times past.") On the subject of these "local action of syphilis," the author keeps very much the old track, and we notice little that claims special attention.

The views entertained by Dr. Wilson on *congenital* and *hereditary* syphilis, we have already adverted to as extreme. That "the transmission of the poison of syphilis from parent to offspring is a fact so well established that no argument can be necessary to give weight to its truth"—we entirely admit. But the assertion, "that the syphilitic poison once received into the blood remains for life,"—after its pathognomonic symptoms are apparently cured—and that it is transmitted hereditarily from generation to generation, we cannot but characterize as an opinion unsustained by facts, and fraught with ill tendencies.

On the subject of treatment, Dr. Wilson's views appear to us to comprise a thoroughly rational and judicious scheme. The *abortive treatment* he of course lays down as the only alternative, as soon as possible after the development of a suspicious pimple or sore upon the organs of generation. In the selection of a caustic application, he gives the preference to potassa, or potassa cum calce, (Vienna paste.) Nitrate of silver, or nitric acid, he considers perfectly useless; "the morbid tissues are not confined to the actual surface, they extend probably to the depth of a line, and the nitrate of silver affects the surface only."

With respect to the constitutional treatment of primary syphilis, Dr. Wilson very reasonably holds, that, if the abortive treatment have been effectually carried out, so soon as the effects of contagion become evident on the organs of generation, there is no longer any poison, and no necessity for internal treatment. As it rarely happens, however, that the surgeon is called upon to treat a venereal sore, until it is several days old, there is more or less chance that the poison has infected the blood, and proper means must be taken to expel it from the system. Mercury is the chief agent to accomplish this, "the great antidote of syphilis," "and for the simple reason," according to Dr. Wilson, "that mercury alone possesses the power of acting upon all the emunctories of the body." Probably, also, it exercises a direct antagonistic or antidotical action upon the poison in the blood.

The author does not urge a mercurial course to the point of salivation; he would, however, keep up a gentle mercurial action for some days after the healing of the ordinary primary sore. In the case of the indurated sore, it must be continued as long as any induration remains; the indurated sore being "a local disease, plus a constitutional disease, a secondary action superadded to a primary disease." To the inflammatory and phagedænic sores, mercury is inapplicable; they must be treated according to the common principles of surgical medicine.

The constitutional treatment of common primary sores may be considered rather as preventive of absorption than as curative. "In constitutional syphilis, however, we have no longer to deal with a possibility of absorption of the poison; the poison is in the blood, and our treatment must be directed to its removal." A mercurial treatment is here indispensable, though dietetic and medicinal adjuvants are not to be lost sight of. Dr. Wilson usually selects the biniodide of mercury in the treatment of constitutional syphilis, giving blue pill, however, the preference in the primary form. He bears testimony to the value of sarsaparilla as a remedy well suited to act on the depurating organs.

A single attack of secondary symptoms does not exhaust the manifestation of the presence of the syphilitic poison in the blood. But "as the attacks of constitutional syphilis become further removed from the original contagion, that is, as the poison becomes more and more assimilated, mercury seems to lose its influence." In a second attack, the biniodide of mercury may retain its power; in the third, the bichloride is prescribed; but in the fourth and successive attacks, the iodide of potassium. In the *chronic* or "tertiary" forms, "mercury is not only inadequate to the removal and cure of the disease, but is actually injurious; it is at this period that iodide of potassium takes the lead as an anti-syphilitic remedy." The nervous complications, syphilitic neuralgia and rheumatism, "yield rapidly and completely" to this medicine; and for affections of a still later period, involving deeper structures of the body, as the fibrous tissue of the penis, the periosteum and bones, it is emphatically the resource. The iodic remedies are not, however, to be pushed after they irritate the system.

We pass over the author's local treatment of the syphilodermata. In congenital syphilis, he considers "the safest practice to be to give mercury to the mother as well as the infant; taking care to moderate the dose to such a degree as not to check or injure the secretion of milk."

On the subject of hereditary syphilis, his views of treatment are in consonance with his pathological doctrines.

"After the age of infancy, congenital syphilis gradually merges into what may be termed hereditary syphilis. The infantile syphilis gets well, but several months or a year afterwards, it breaks out again. Sometimes, however, the patient has been free from any indications of syphilis in his infantile age, the first manifestations of its presence in the system being delayed to the period of advanced childhood, puberty, or adult life. This more properly constitutes hereditary syphilis.

The kind of syphilitic disease now under consideration, in its more recent forms yields without much difficulty to the bichloride of mercury; when more advanced, the iodide of potassium is a useful auxiliary; and in a more distant remove, the combinations of iodine, mercury, arsenic, and cod-liver oil, become valuable remedies."

For the Esthiomenous affections of the skin, which Dr. Wilson attempts to trace "to the syphilitic poison in a distant remove from its original source,"—"a class of diseases essentially refractory,"—he claims the discovery of a correct principle of treatment; "the treatment by alterative doses of mercury." In his hands,

"This treatment has been most encouraging and most successful. They have yielded before the antisyphilitic treatment, as if 'syphilis' were stamped upon them in printed characters; and they certainly belong to that category of diseases which, treated by syphilitic remedies, are to be relieved and cured; but which treated in any other way are not only incurable but often destructive of life."

We have perhaps been somewhat prodigal of space in our abstract of this book. But it is certainly a very good resumé of received opinions on syphilis, and presents many original and striking views on the subject.

